# Cellular mechanism of gain-of-function mutation I173M in sheep *MC4R* gene identified in year-round and seasonal estrus breeds through whole-genome resequencing

**DOI:** 10.1186/s12711-026-01091-4

**Published:** 2026-10-12

**Authors:** Yuta Yang, Yuxin Kang, Peiyao Liu, Chunna Cao, Yang Li, Ran Li, Xianyong Lan, Chuanying Pan

**Affiliations:** College of Animal Science and Technology, Northwest A&F University, Yangling, Shaanxi 712100 China

## Abstract

**Background:**

Investigating key genes regulating year-round estrus can improve sheep reproductive performance and industry efficiency. Although several candidate genes have been proposed, the key regulators underlying estrus seasonality remain unclear. Therefore, this study aimed to identify critical genetic variants associated with year-round estrus using whole-genome resequencing data from multiple sheep breeds.

**Results:**

In this study, we analyzed whole-genome resequencing data from 392 sheep across six year-round and ten seasonal estrus breeds, and identified the Melanocortin 4 receptor (*MC4R*) gene as a key regulator of estrus seasonality. A missense mutation in the *MC4R* gene, I173M (g.59480440G > C; p.Ile173Met), was significantly enriched in year-round estrus breeds. Transcriptome analysis following *MC4R* I173M transfection revealed differential expression of genes enriched in reproductive pathways. Subsequent functional experiments demonstrated that the I173M mutation increased *MC4R* constitutive activity, activated cAMP and MAPK/ERK signaling, and promoted both granulosa cell proliferation and the expression of steroidogenic regulatory genes. Cells harboring the I173M mutation exhibited substantially amplified transcription levels of gonadotropin signaling components. Specifically, follicle-stimulating hormone receptor (*FSHR*) and luteinizing hormone receptor (*LHR*) were expressed at much higher levels relative to wild-type (WT) controls.

**Conclusions:**

Our study provides evidence that *MC4R* functions as a molecular modulator of estrous cyclicity, with the I173M missense mutation identified as a critical functional variant driving constitutive estrous activity in ovine species.

**Supplementary Information:**

The online version contains supplementary material available at https://doi.org/10.1186/s12711-026-01091-4.

## Background

Lamb and mutton play a vital role in the global meat industry, with recent years witnessing a substantial increase in production. However, ongoing shortages in supply have driven up prices, highlighting the imbalance between growing consumer demand and limited domestic production capacity. To address this disparity, it becomes crucial to prioritize the enhancement of the reproductive performance of ewes to bolster lamb and mutton production.

Mammals typically exhibit a seasonal reproductive pattern, leading to a pronounced imbalance in the supply of animal products throughout the year [1, 2]. Seasonal estrus in sheep serves as a classic example of this phenomenon. Mammalian estrus can be classified into short-day breeders and long-day breeders, with sheep categorized as short-day breeders, primarily displaying estrus during the autumn and winter seasons [3]. While this seasonal estrus ensures favorable environmental conditions for offspring survival, it also results in an extended anestrous period, challenging successful mating and diminishing reproductive efficiency [4]. Reproductive efficiency stands as a critical factor in the economic viability of sheep farming, yet the seasonality of estrus remains a pivotal constraint to the industry’s progress. The estrous cycle is a crucial factor influencing the reproductive efficiency of sheep. Year-round estrus, characterized by the absence of seasonal constraints, represents a substantial enhancement in production efficiency. Consequently, the exploration of key genes associated with seasonal estrus and the investigation of the molecular mechanisms underpinning seasonal estrus in sheep are of paramount importance for augmenting their reproductive capabilities.

Sheep reproduction is primarily regulated by the Pineal-Hypothalamus-Pituitary-Ovary (PHPO) axis, with its secreted reproductive hormones exhibiting seasonal fluctuations, influenced by photoperiodic changes (seasonal variations) [5–8]. In sheep, light signals received by the retina trigger changes in melatonin secretion within the pineal gland, initiating a regulatory process involving melatonin receptors and influencing the regulation of the Thyroid-Stimulating Hormone (TSH)-Dio and *KISS1*-*GPR54* receptor (kisspeptin-*GPR54*) systems [3, 9]. This regulatory process, achieved through the modulation of hormone secretion, ultimately governs reproductive activities. Due to the seasonality of estrus, several studies have suggested that circadian rhythm genes may influence the seasonal reproduction of sheep [10]. This includes genes such as *Clock*, *BMAL1*, *Per1*, *Per2*, *Cry1* and *Cry2*, which are believed to potentially affect seasonal estrus [11, 12]. Transcriptomic sequencing has been employed to analyze tissues, for instance, the hypothalamus, pituitary, and ovary tissues of seasonal and non-seasonal reproductive sheep [13–15], revealing candidate genes such as *MTNR1A* [16], *GNAQ* [17] and *SEMA7A* [14] linked to sheep estrus. These genes may play crucial roles in regulating the seasonal reproductive processes in sheep, offering valuable clues for unraveling the mechanisms behind seasonal reproduction. However, key genes affecting the seasonal characteristics of estrus in sheep have not yet been identified. The manifestation of year-round estrus in certain sheep breeds is a consequence of rigorous human selection, possibly driven by the selection for specific major-effect genes and critical mutations. In sheep, breeds such as Hu sheep, Large Tail Han sheep, and Cele black exhibit year-round reproductive behaviors [18–21]. In contrast, Tibetan sheep and Tan sheep develop their gonads at specific times of the year and demonstrate seasonal reproductive behaviors [5, 16]. Therefore, using both year-round and seasonal estrus sheep breeds as models can facilitate a more comprehensive investigation into the molecular mechanisms underlying seasonal reproduction in sheep. Genomic research offers a new approach by establishing a direct link between phenotypes and genetics, providing a fresh perspective on the study of complex traits.

In this study, fixation index (FST) and genome-wide association study (GWAS) methods were used to identify the key gene melanocortin 4 receptor (*MC4R*), located on sheep chromosome 23, and its I173M (g.59480440G > C, p.Ile173Met) mutation, which are associated with year-round estrus in sheep. The effects of this mutation on MC4R protein expression, membrane localization, and transcriptional regulation were further investigated. Subsequent experiments, based on RNA sequencing data, confirmed the impact on the downstream cAMP and MAPK/ERK signaling pathways of I173M. Importantly, this study represents the first functional investigation of the *MC4R* I173M variant in sheep, providing evidence of its role in modulating estrous cyclicity through effects on receptor activity, signaling pathways, and granulosa cell function. Taken together, these findings led us to hypothesize that the I173M missense mutation in the *MC4R* gene is a functional mutation affecting receptor signaling and plays a key role in enabling the year-round estrus phenotype observed in selected sheep breeds. Our results underscore the significant role of the *MC4R* gene in year-round estrus in sheep and provide an important reference for exploring the mechanisms of estrus in sheep, as well as for enhancing their reproductive performance.

## Methods

### Animal and sample collection

Whole-genome sequencing (WGS) data from 392 sheep representing 16 breeds were retrieved from the NCBI Sequence Read Archive (SRA) database (https://www.ncbi.nlm.nih.gov/sra/), including 160 individuals from six year-round estrous breeds and 232 individuals from ten seasonally estrous breeds. Detailed sample information is provided in Additional file 1, Table S1, and additional metadata (including sequencing depth and data source) are provided in Additional file 2, Table S2. Sheep breeds were classified as year-round or seasonal breeders based primarily on the reproductive characteristics documented in Animal Genetic Resources in China: Sheep and Goats [22], with additional support from published studies describing reproductive seasonality in representative sheep breeds [19, 20, 23, 24].

### Reads mapping and SNPs calling

The raw FASTQ files underwent quality filtering using the fastp (v0.20.0) with the options “-q 20 -u 20 -n 5 -l 80 -w 8” [25]. BWA software (v0.7.17) was employed to align the genome data to the sheep reference genome (ARS-UI_Ramb_v2.0) [26]. SAMtools software (v1.7) was used to convert SAM files to BAM files [27]. The BAM files were sorted using SAMtools (v1.7), and the duplicates were removed using Picard (v2.20.3) (https://broadinstitute.github.io/picard). Subsequently, the variants calling were called using GATK (version 4.1.7.0) and filtered with the parameters “QD < 2.0, QUAL < 30.0, FS > 60.0, MQ < 40.0, MQRankSum < − 12.5, ReadPosRankSum < − 8.0” [28]. VCFtools (v0.1.16) was used to filter the resulting SNP set, biallelic SNPs with a minimum allele frequency > 0.05 and a missing ratio < 0.1 were retained for subsequent analyses.

### Population structure

Principal component analysis (PCA) and ADMIXTURE were ueds to analyze the population genetic structure. PCA was conducted using the smartpca program in EIGENSOFT v5.0 [29]. ADMIXTURE (version 1.3.0) was utilized with K set from 2 to 10 [30].

### F_ST_ analysis

The genome-wide selection signatures were identified by using FST. We compared the year-round estrus breeds with seasonally estrus breeds to characterize genetic differentiation between these two groups. VCFtools (version 0.1.16) was used to compute F_*ST*_ values with 50 kb window and 10 kb steps. The regions (top 1‰) were considered as signal of positive selection, and these regions were annotated by ANNOVAR [31]. We also calculated the Tajima’s D and PI statistic for 2 groups to strengthen our results by VCFtools [32].

### GWAS analysis

In this study, perennial estrus and seasonal estrus characteristics were treated as binary variables (0 or 1). The EMMA software was used for GWAS analysis [33]. A mixed linear model was used for GWAS analysis: y = Xα + f + ε, where y is the phenotype value, X is the covariate matrix (fixed effects), α is the vector of fixed-effect coefficients, f is the covariance (relationship) matrix, ε is a random effect. PLINK (v1.90b7) was used for preliminary data processing and PCA calculation [34]. The first three principal components (PC1, PC2, PC3) from PCA, explaining 13.639%, 7.322%, and 5.418% of the effect values, were used as corrected fixed effects. The emmax-kin module of EMMA was used to calculate the relationship as a covariance. The calculation was performed using the default parameters of the EMMAX software “emmax-intel64 -v -d 10 -t input -p estrus_data -k input.aIBS.kinf -c covariate.pca -o output”. Then, personal python scripts were employed for result visualization. The whole-genome significance threshold was defined as the Bonferroni test threshold and as 0.05/total SNPs. Subsequently, the SnpEff (V4.2) was used to annotate the functional SNPs [35]. Allele frequencies of SNP loci in each sheep population were calculated using the -freq parameter of VCFtools v3 [32].

### In vitro mutagenesis of *MC4R*

The coding regions of wild-type (WT) and mutant sheep *MC4R* were sub-cloned into the pcDNA3.1 (+) vector from Invitrogen (Carlsbad, CA, USA). The WT and M173 3×Myc-tagged *MC4R* vectors were synthesized by Nanjing GenScript Corporation. Plasmids for transfection were prepared using the Endofree Plasmid Maxi Kit from TianGen Biotech. DNA sequencing was conducted to verify the correctness of the full coding region.

### Cell culture and luciferase reporter assay

This study utilized the luciferase reporter system to investigate the activation of downstream signals through *MC4R* by two ligands. The luciferase reporter vectors employed were pGL4.29 from Promega (Madison, WI, USA), containing cAMP response element (CRE) in the promoter regions for monitoring cAMP activation, and pGL4.33 plasmid with serum response element (SRE) sequences for detecting MAPK/ERK signaling pathway activation [36, 37]. Human embryonic kidney (HEK) 293T cells were obtained from the Cell Bank of the Chinese Academy of Sciences (Shanghai, China) and were authenticated by the supplier. Cells were grown at 5% CO_2_ in DMEM supplemented with 10% fetal bovine serum, and 100 units/ml of penicillin and 100 µg/ml streptomycin. The brief operating procedure was as follows: the HEK293T cells were passaged to a 6-well plate 24 h before transfection. Then, a mixture containing 1000 ng luciferase reporter vector, 500ng WT or mutant sheep *MC4R* expression plasmid (or empty pcDNA3.1 plasmid), 300ng pEGFP-N1 (as the internal control for transfection normalization), and 4µL PEI transfection reagent (Fusheng Biotechnology, Shanghai, China) was used to transfect these cells. The transfected cells continued to grow in the original medium for 24 h, then they were pipetted down and transferred to a 48-well plate to grow for another 24 h to reach a density of 2 × 10^5^ cells per well. The agonists α-MSH and β-MSH were obtained from GenScript Biotechnology (Nanjing, China). The α-MSH and β-MSH were diluted to working concentration in serum-free medium, and then added to the 48-well plate to treat cells for 6 h. After processing, the cells were lysed with 1× passive lysis buffer (YEASEN, Shanghai, China), and the luciferase substrate was added for reaction. For each assay, two additional 48-well plates (*n* = 3) were used as technical replicates and data were shown as mean ± SD.

### Cell transfection and real-time quantitative PCR (RT-qPCR)

To functionally characterize the regulatory roles of WT and mutant *MC4R* in ovine reproductive physiology, we conducted plasmid transfection studies on sheep primary ovarian granulosa cells derived from 5-month-old Hu sheep (Lingjun Biotechnology, Hangzhou, China) using WT *MC4R* or its M173 missense variant cloned into pcDNA3.1 vectors. Cells were cultured in DMEM/F12 medium (Hyclone) supplemented with 20% FBS (Gibco), penicillin (100 U/mL), and streptomycin (100 µg/mL) under standard conditions (37 °C, 5% CO₂). At 70–80% confluency, transfection was performed with Lipofectamine 3000 (Invitrogen) following manufacturer protocols, employing three experimental groups: (1) WT *MC4R*-pcDNA3.1, (2) M173 *MC4R*-pcDNA3.1, and (3) empty pcDNA3.1 control. Cells were harvested 48 h post-transfection for transcriptomic profiling, including RNA extraction and quantitative analysis of steroidogenic pathway gene expression.

Total RNA was extracted from the cells using RNAiso Plus reagent (Takara, Beijing, China) following the manufacturer’s protocol. Subsequently, RNA was reverse transcribed using the Hifair^®^ III Reverse Transcriptase kit (Yeason, Shanghai) according to the manufacturer’s instructions. RT-qPCR was performed using ChamQ Universal SYBR qPCR Master Mix (Vazyme, Nanjing) on an ABI QuantStudio 5 real-time PCR system (ABI, USA). GAPDH was used as the reference gene, and gene expression data were normalized using the 2^−ΔΔCT^ method. The information for the quantitative primers is provided in Additional file 3, Table 3.

### Cell proliferation assays

Cell viability was assessed using the CCK-8 kit (Beyotime, Shanghai, China). After transfection, cells were cultured in 48-well plates, and viability was evaluated at 24 h and 48 h. According to the manufacturer’s instructions, 10 µL of CCK-8 solution was added to each well 2 h before measurement, followed by incubation at 37 °C in a 5% CO₂ environment. Absorbance at 450 nm was recorded using a fluorescence multimode microplate reader (BioTek, Winooski, VT, USA). Cell proliferation was assessed using the EdU assay kit (Beyotime, Shanghai, China). Cells were seeded in 48-well plates, fixed 48 h post-transfection, and stained with the BeyoClick™ EdU-555 kit according to the manufacturer’s instructions. Fluorescence images were captured using a microscope, and statistical analysis was performed with ImageJ software. All experiments were conducted in triplicate.

### Western blot

After transfecting HEK293T cells with WT or M173 *MC4R* plasmids (or empty pcDNA3.1 plasmid) for 24 h, cells were stimulated with 10^− 5^M α-MSH for 15 min. The control group was treated with serum-free medium for 15 min. The cultured cells were lysed in RIPA buffer containing 1% PMSF and 2% phosphatase inhibitors. After centrifugation, the supernatant was collected, mixed with 4× protein loading buffer at a 3:1 ratio, and subjected to protein denaturation at 100℃ in a constant temperature metal bath for 10 min. Following SDS-PAGE gel electrophoresis separation of 20ng protein samples, the proteins were wet-transferred onto a PVDF membrane. The membrane was then blocked at room temperature for 2 h using a 5% skim milk solution. Primary antibodies, Myc (1:2000) and β-actin (1:5000), were incubated overnight at 4℃. After washing with TBST for 3 h, each wash lasting 5 min, the membrane was incubated with secondary antibodies (1:10000) for 2 h on a shaker. Subsequent to another 3 washes with TBST, chemiluminescent ECL reagent was applied, and the membrane was scanned using a chemiluminescence imager. ImageJ software was used for grayscale analysis of bands, with relative expression levels of the target bands calculated by comparing grayscale values with β-actin protein bands.

### Immunofluorescence

HEK293T cells were seeded onto glass coverslips in 12-well plates and transfected with 500 ng of WT or M173 *MC4R* plasmids. After 24 h of transfection, the cells were fixed with 4% formaldehyde in PBS for 10 min at room temperature. They were then washed three times for 5 min each with phosphate-buffered saline (PBS). Next, the cells were permeabilized with 0.1% Triton X-100 in PBS for 5 min. Following permeabilization, a blocking step was performed by incubating the cells with 5% bovine serum albumin (BSA) for 1 h. After blocking, the cells were incubated overnight at 4 °C with Myc-Tag (19C2) Mouse mAb antibody (Abmart) at a dilution of 1:200 in BondTM primary antibody diluent. The next day, the cells were washed with PBS to remove unbound primary antibodies. Then, they were incubated with a fluorescently labeled secondary antibody, iFluor 488 (HA1125), at a dilution of 1:500. This incubation was performed in a dark environment for 2 h. After the secondary antibody incubation, the cells were washed to remove unbound secondary antibodies. Finally, Hoechst 33,342 dye was added for nuclear staining, and the cells were incubated with the dye for 20 min. Slides were imaged using a confocal microscope and images processed using FIJI. Results are from three independent experiments.

The immunofluorescence identification method for ovarian granulosa cells was performed following the same protocol as described above. The FSHR antibody (Proteintech, Cat. No. 22665-1-AP) and the fluorescence-labeled secondary antibody, Alexa Fluor 594-conjugated Affinipure Goat Anti-Mouse IgG (H + L), were both purchased from Proteintech.

### Flow cytometry

The flow cytometry assay to quantitate the expression levels of *MC4R* involved the following steps: Cells were plated into a six-well plate and prepared by washing with PBS. The cells were then fixed with 4% paraformaldehyde and incubated with a blocking solution (5% BSA). After blocking, the cells were incubated with Myc-Tag (19C2) Mouse mAb antibody (dilution 1:200) for 1 h. Subsequently, the cells were washed and incubated with a fluorescently labeled secondary antibody, iFluor 488 (dilution 1:500) for 2 h. Flow cytometry analysis was performed using a C6 flow cytometer to measure the fluorescence emitted by the cells. Unstained cells and secondary antibody–only controls were included to assess autofluorescence and non-specific binding. The expression levels of the *MC4R* variants were calculated as a percentile of WT expression using a specific formula [38]. The entire assay was conducted at room temperature.

Samples for apoptosis detection were collected 48 h post-transfection, digested, centrifuged, and washed with PBS. Subsequently, 5 µl Annexin V/FITC (Solarbio, CA1020) was added, mixed gently, and incubated at room temperature for 5 min in the dark. The samples were then analyzed using flow cytometry (CytoFLEX, Beckman Coulter, USA). Cell apoptosis was quantified with FlowJo_V10 software.

### RNA-sequencing and bioinformatic analyses

HEK293T cells were seeded onto glass coverslips in plates and transfected with WT or M173 *MC4R* constructs for transcriptome sequencing. Total RNA was extracted using TRIzol (Invitrogen) following the standard protocol. Transcriptome sequencing was performed by Guangzhou GENE DENOVO Biotechnology Co., Ltd. Clean reads were aligned to the reference genome using HISAT2, followed by transcript reconstruction using StringTie. Gene expression levels were quantified using RSEM based on the aligned reads. Differentially expressed genes (DEGs) between the WT and M173 groups were identified using the DESeq2 package based on read count data, with an FDR < 0.05 and an absolute log₂ fold change (|log₂FC|) > 2 [39]. Gene Ontology (GO) and Kyoto Encyclopedia of Genes and Genomes (KEGG) pathway annotation and enrichment analyses were conducted using the Gene Ontology database and the KEGG pathway database, respectively.

### Statistical analysis

All statistical analyses were performed using SPSS software (version 23.0; IBM, Chicago, IL, USA). For the half-maximal effective concentration (EC_50_) data, repeated-measures ANOVA was conducted, followed by nonlinear regression analysis using Prism software version 4 (GraphPad Software). One-way ANOVA was applied to analyze cell viability data, and Duncan’s multiple range test was subsequently performed for pairwise comparisons. Other datasets were analyzed using Student’s t-test. Continuous variables are reported as mean ± SD. All experiments included at least three independent biological replicates, a *P*-value < 0.05 was considered statistically significant.

## Results

### The screening of candidate genes for year-round estrus in sheep

**Fig. 1:**
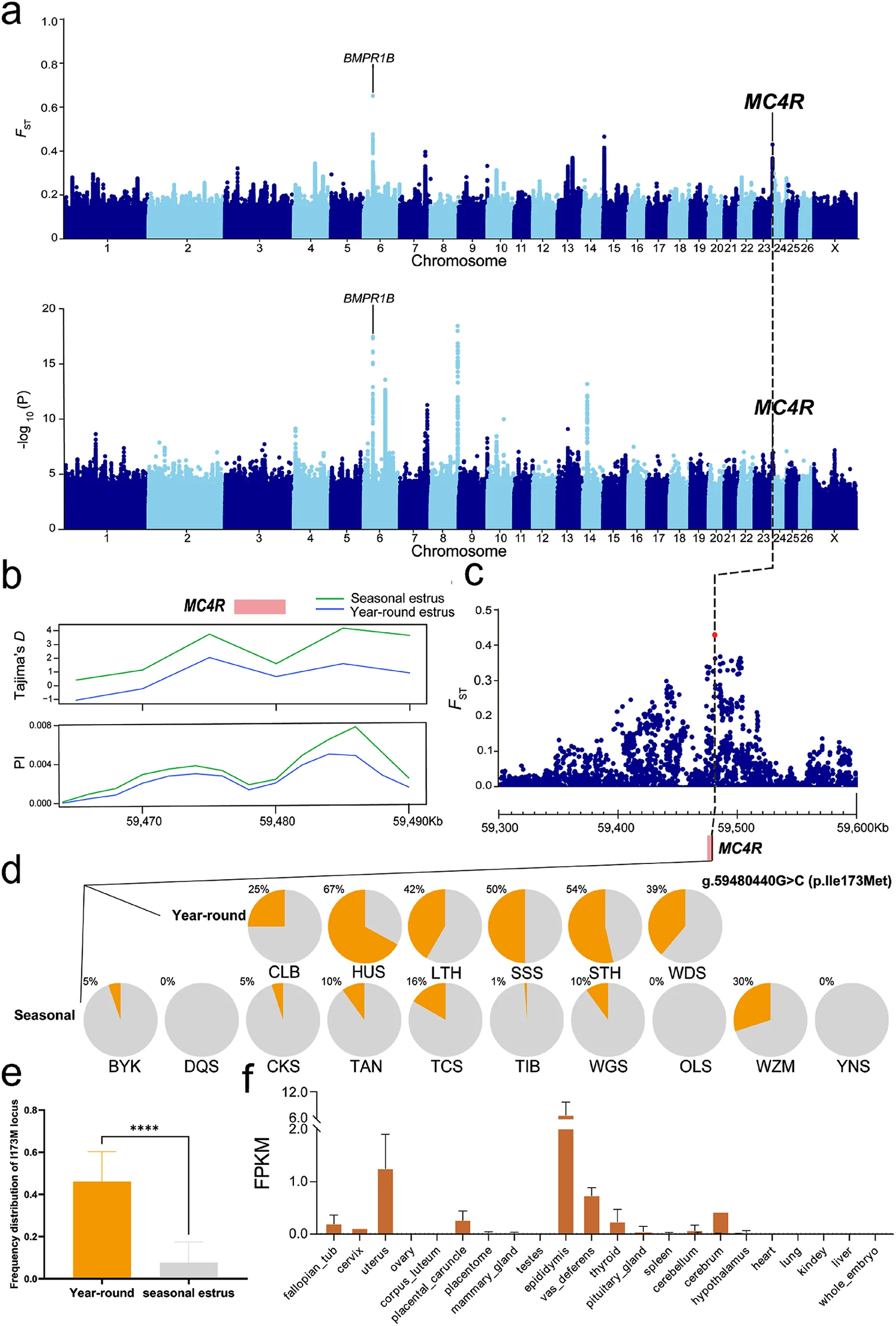
The *MC4R* I173M (g.59480440G > C, P.Ile173Met) variant as a candidate locus for year-round estrus in sheep. (**a**) Manhattan plots of selective signatures with *F*_ST_ and GWAS. (**b**) PI and Tajima’s D around *MC4R* in seasonal (green) and year-round (blue) estrus breeds. (**c**) The *F*_ST_ result of the region (chr23:59,300 − 59,600Kb) showed that I173M locus was the peak site. (**d**) Frequency distribution of the mutant allele in seasonal and year-round estrus sheep breeds. (**e**) Allele frequency differences at the I173M locus between seasonal and year-round estrus sheep breeds. (**f**) Expression of *MC4R* gene in sheep different tissues. Data source: http://animal.omics.pro/code/index.php/RGD

To identify selection signals associated with year-round estrus in sheep, this study utilized whole-genome resequencing data from 6 year-round estrus breeds and 10 seasonal estrus breeds, encompassing a total of 392 sheep. The PCA results revealed a well-defined clustering of individuals within each breed, indicating the suitability of these data for subsequent analysis (Additional file 4, Fig. S1a). ADMIXTURE analysis identified ancestry components across all individuals. At K = 3, Tibetan and Yunnan sheep were clearly differentiated from other domestic breeds (Additional file 5, Fig. S2), in agreement with the PCA results. The model with K = 5 exhibited the lowest CV error. *F*_ST_ and GWAS analysis were conducted between the year-round estrus and seasonal estrus breeds. Gene annotation of the top 1‰ significant loci revealed genes associated with lambing (*BMPR1B*) and year-round/seasonal estrus (Melanocortin 4 receptor, *MC4R*) (Fig. 1a). The complete lists of annotated genes from GWAS and FST are provided in Additional file 6, Table S5 and Additional file 7, Table S6, respectively. Notably, a highly selected region in the *MC4R* gene was discovered both using *F*_ST_ and GWAS analysis, which was also confirmed by nucleotide diversity (PI) and Tajima’s *D* results in this region (Fig. 1b). These results suggest that the *MC4R* gene may be involved in the regulation of estrus seasonality in sheep, and this gene has also been reported to play a role in the regulation of the estrous cycle in animals [40]. The missense mutation I173M (g.59480440G > C, P.Ile173Met) was the strongest selected site in the *MC4R* gene (Fig. 1c). The mutant allele was significantly more frequent in year-round estrus breeds than in seasonal estrus breeds (*P* < 0.0001), indicating a strong association with year-round estrus (Fig. 1d and e). The expression of the *MC4R* gene in various tissues of sheep was comparatively analyzed using the Animal Genetics and Genomics Database (http://animal.omics.pro). The results revealed that *MC4R* is expressed in reproductive-related tissues, such as the epididymis, oviduct, vas deferens, uterus, cervix, and thyroid (Fig. 1f). Additionally, we scanned SNPs in the *MC4R* gene across the 16 sheep breeds. Detailed SNP information and allele frequencies are provided in Additional file 8, Table S6 and Additional file 9, Table S7, respectively, and the frequency distribution is visualized as a heatmap in Additional file 4, Fig. S1b.

### Conservation and mutation site analysis of the candidate gene MC4R associated with estrus seasonality in sheep

**Fig. 2:**
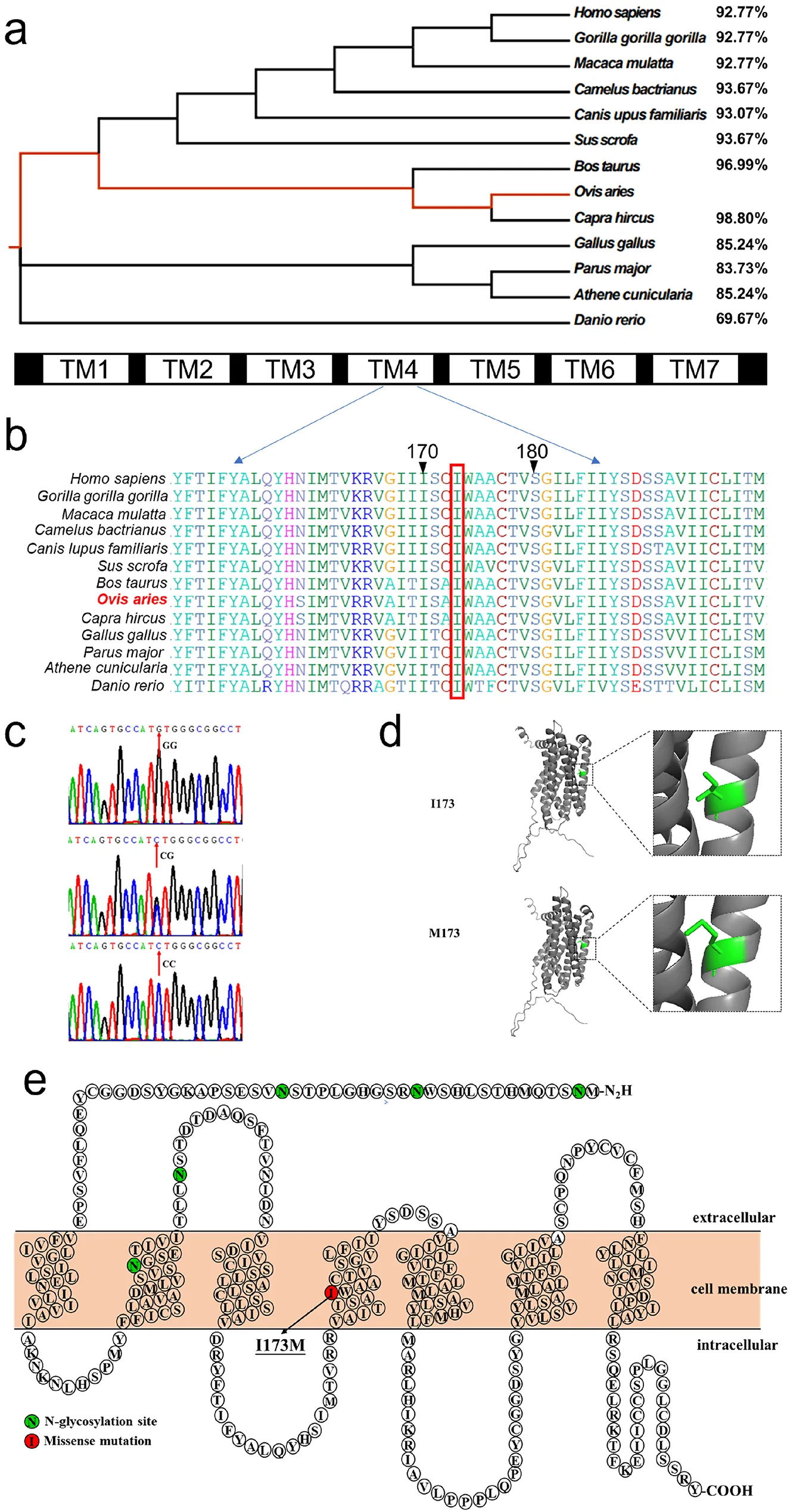
Structural and evolutionary analysis of the *MC4R* I173M variant in sheep. (**a**) The conservation analysis of the MC4R protein. (**b**) Sequence alignment of the amino acid at position 173 in 13 species. (**c**) DNA-sequencing electropherograms of the I173M mutation. (**d**) Three-dimensional protein structure changes of I173M before and after mutation introduction. (**e**) MC4R protein highlighting amino acids affected by I173M variant. (http://wlab.ethz.ch/protter/start/)

The results of the comparative analysis of the MC4R protein in 13 vertebrate species indicate that MC4R is highly conserved across different species, with conservation levels exceeding 90% in animals such as goats, sheep, and pigs. Additionally, the similarity between sheep MC4R protein and human MC4R protein is 92.77% (Fig. 2a). The analysis of the conservation of the Ile amino acid at position 173 in the sheep MC4R across different species showed that this site was highly conserved, indicated that mutation at this position may have an impact on protein function (Fig. 2b). Subsequently, we employed PCR primers for *MC4R* amplification (F: TCAGTCAGTCCAGAGGGGAC, R: TGTGTTTAGCATCGCGTTTG) to validate the sequenced site. The sequencing results for the mutation site are shown in the Fig. 2c. The PyMol v7.0.1 software was used for protein three-dimensional structure prediction of the missense mutation. Introducing the M173 amino acid revealed a change in the three-dimensional structure of the MC4R protein (Fig. 2d). This suggests that the mutation may impact the function of the MC4R protein. Further prediction through the online tool (http://wlab.ethz.ch/protter/start) indicated that the missense mutation g.59480440G > C is located in the fourth transmembrane domain (TM4) of the MC4R protein (Fig. 2e). Previous studies have demonstrated the crucial importance of the third and fourth transmembrane domains of *MC4R* for its binding [41]. Hence, it is inferred that this mutation may potentially impact the functionality of receptor.

### The I173M mutation in sheep does not impact the localization and expression of MC4R

**Fig. 3:**
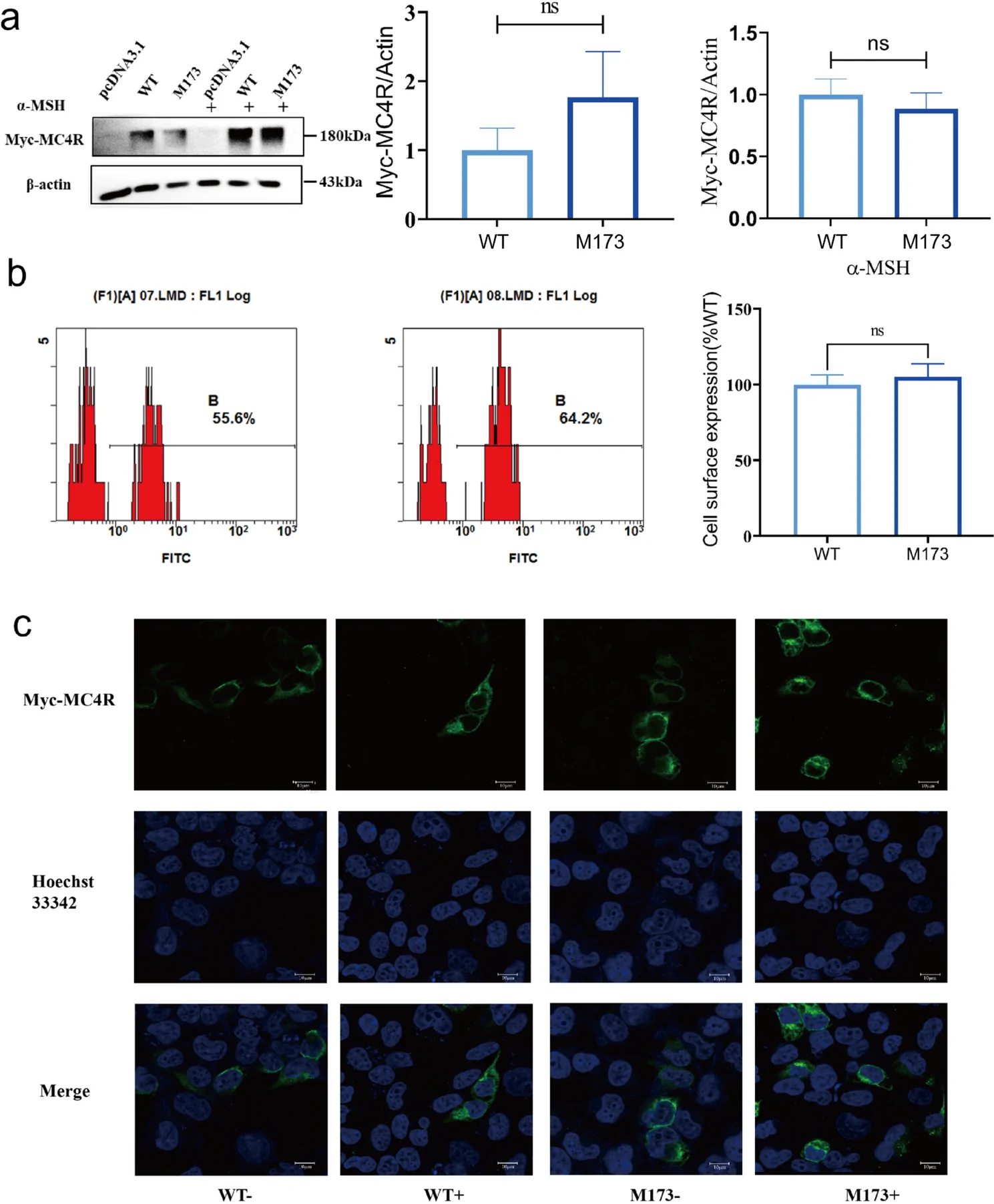
Functional effects of the *MC4R* I173M mutation on receptor activity and localization. (**a**) Western bloting results of WT and M173 MC4R before (-) and after (+) stimulation by α-MSH. (**b**) Flow cytometry results of the expression of WT and M173 receptors (*n* = 3). (**c**) Confocal imaging of membrane localization of WT and I173M MC4R. Cells were stained with anti-Myc antibody before and after Triton permeabilization (+), nuclei are shown in blue (scale bar: 10 μm)

To investigate whether the I173M mutation alters the expression of MC4R on the cell membrane, we transfected HEK293T cells with WT and M173 recombinant vectors and assessed the protein expression level of MC4R. The electrophoresis results demonstrated that the I173M mutation does not affect the expression level of MC4R protein (Fig. 3a). To further evaluate the impact of the mutation on MC4R expression, we used flow cytometry to detect the surface expression of WT and M173 proteins in HEK293T cells. There was no significant difference in the expression levels of WT and M173 receptors on the cell membrane (*P* > 0.05, Fig. 3b). Previous studies have shown that MC4R is localized on the cell membrane and undergoes continuous endocytosis and recycling [42]. Immunostaining of HEK293T cells expressing the two recombinant plasmids revealed that both WT and M173 *MC4R*s are normally expressed on the cell membrane without permeabilization. After permeabilization, both receptors were successfully transported to the intracellular membrane, indicating that the mutation does not affect receptor localization and trafficking on the cell membrane (Fig. 3c). These results suggest that the I173M mutation does not significantly alter the expression or localization of *MC4R* on the cell membrane.

### The transcriptional regulation of sheep *MC4R* I173M mutant

**Fig. 4:**
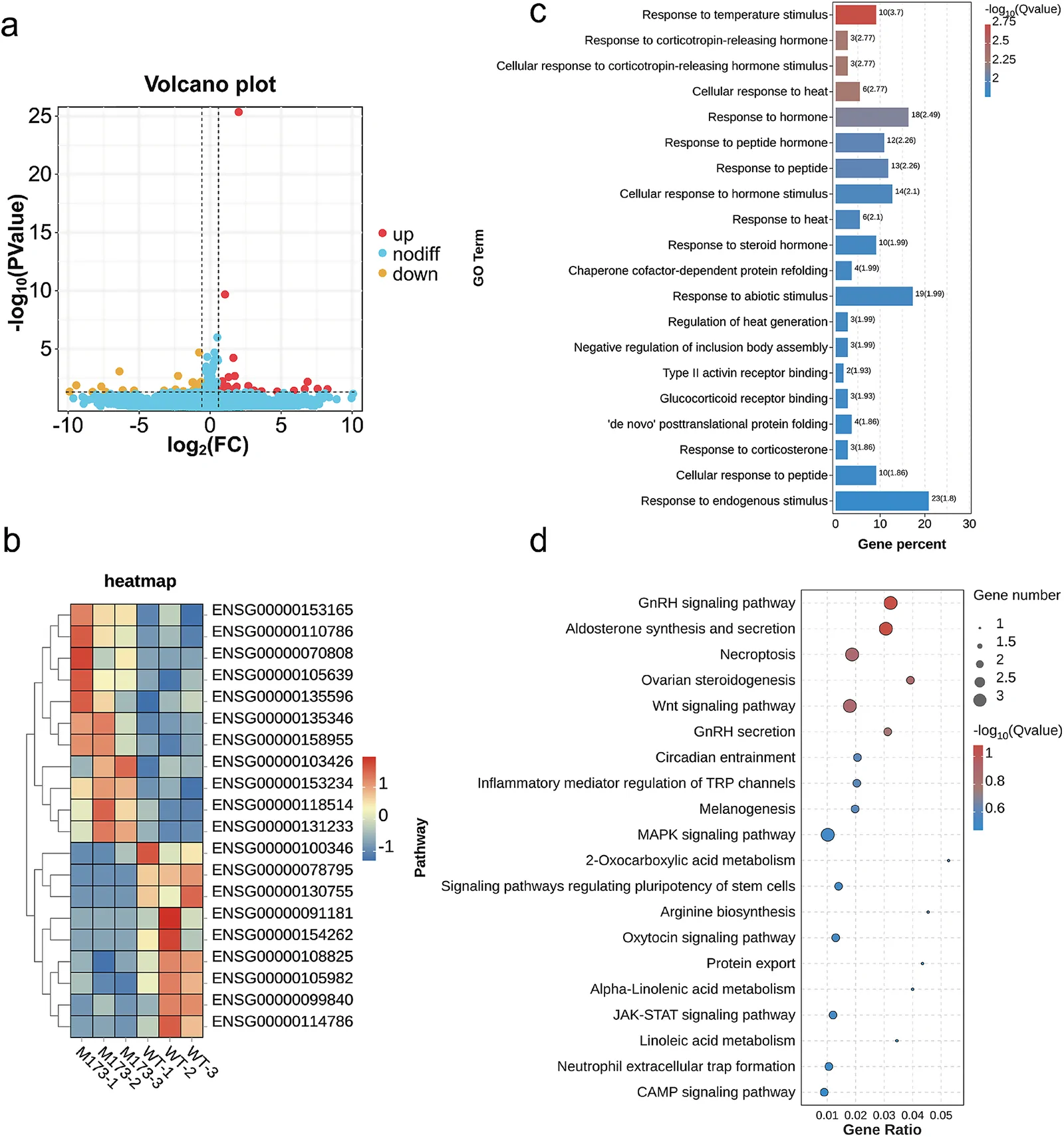
RNA sequencing to analyze the regulation of I173M mutation on downstream signaling pathways (*n* = 3 per group). (**a**) Volcano plots: differentially expressed genes (DEGs) of WT and M173 *MC4R*s. (**b**) Heat map: differentially expressed genes (DEGs). (**c**) GO analysis of differentially DEGs. (**d**) KEGG pathway analysis

Subsequently, WT and M173 *MC4R* plasmids were transfected into HEK293T cells, followed by sample collection for transcriptome sequencing to investigate DEGs and their associated pathways. After filtering the transcriptome sequencing results, 53 DEGs were identified, with 26 significantly upregulated and 27 significantly downregulated genes (Fig. 4a). Among them, genes such as *NR4A2*, *IL5RA*, and *CAMK2A* showed upregulation in cells transfected with the M173 plasmid (Fig. 4b). GO analysis results indicated that DEGs were enriched in biological processes such as aminomuconate-semialdehyde dehydrogenase activity, interleukin-5 receptor activity, follicle-stimulating hormone secretion, signaling receptor ligand activity and receptor activator activity (Fig. 4c). Using the KEGG database for pathway enrichment analysis of DEGs, the most significantly enriched pathways included the GnRH signaling pathway, Aldosterone synthesis and secretion, Necroptosis, Ovarian steroidogenesis, Wnt signaling pathway (Fig. 4d). It is worth noting that the cAMP signaling pathway and MAPK signaling pathway of *MC4R* are also significantly enriched (Fig. 4d). Certainly, follow-up studies can be undertaken to delve deeper into the implications of these DEGs and pathways. Here, we mainly focus on the regulation of cAMP signaling pathway and MAPK signaling pathway by the M173 *MC4R* receptor.

### The I173M mutation amplifies the constitutive activity and activates cAMP signaling in the MC4R receptor

*MC4R* mainly couples to the G-protein G_S_ and it is well-established that binding of Pro-opiomelanocortin (POMC)-derived peptides (α-/β-MSH [melanocyte-stimulating hormone]) to membrane-bound *MC4R* activates G proteins (Gαs) and stimulates the production of cyclic AMP (cAMP) [43, 44].

**Fig. 5:**
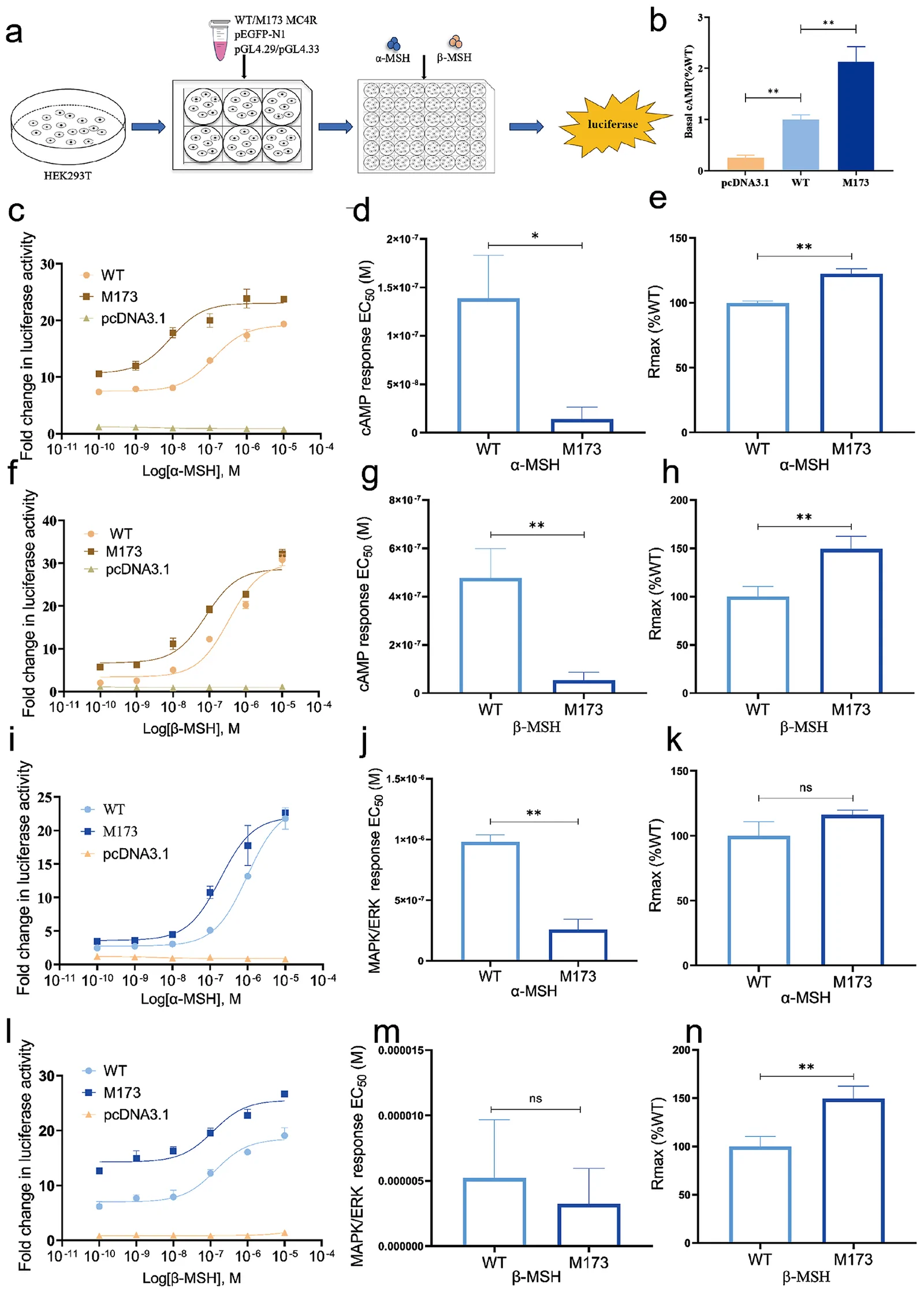
Effects of the I173M mutation on constitutive activity and activation of *MC4R* signaling pathways. (**a**) Experimental protocol. (**b**-**h**) Basal and α-MSH/β-MSH–stimulated cAMP signaling in WT and I173M *MC4Rs*, including EC_50_ and Rmax values. (**i**-**n**) α-MSH/β-MSH–stimulated MAPK/ERK signaling in WT and I173M *MC4Rs*, including EC_50_ and Rmax values. *P < 0.05, **P < 0.01. Data represent mean ± SD of three replicates (*n* = 3)

Wild-type *MC4R* exhibits basal (constitutive) activity, and naturally occurring mutations have been identified that result in either increased or decreased basal activities [45]. The transfection of HEK293T cells with pGL4.29, pEGFP-N1, and WT/M173 *MC4R*, followed by the evaluation of constitutive activity (cAMP basal level) using a dual luciferase reporter gene assay in the absence of agonist stimulation (Fig. 5a), revealed a significant increase in cAMP basal level by the WT *MC4R* receptor compared to pcDNA3.1 (*P* < 0.05, Fig. 5b). Moreover, the M173 mutant receptor exhibited significantly increased basal activity compared to the WT receptor (*P* < 0.05). Subsequent evaluation of the signal transduction capacity of the receptors before and after mutation involved stimulating cells expressing recombinant WT and M173 *MC4R* vectors with six concentrations of α-MSH/β-MSH (ranging from 10^− 10^ to 10^− 5^) and assessing receptor signal transduction ability using a dual luciferase reporter gene assay (Fig. 5a). The data obtained from concentration-response experiments (all concentration-response curves for two receptors and the two ligands are presented in Fig. 5c and f) were analyzed using fitting non-linear regression models to determine the response value of each ligand at the receptor mutation (Rmax) and the potency of the ligand at the receptor mutation (EC_50_). The cAMP activity of the M173 *MC4R* increased fourfold compared to that of WT *MC4R* upon stimulation with the α-MSH ligand (Fig. 5d), and the Rmax of M173 receptor to cAMP increased significantly (*P* < 0.01, Fig. 5e; Table 1). After using β-MSH stimulation, the results were found to be consistent with those of α-MSH stimulation (Fig. 5g and h). Specifically, the EC_50_ value of β-MSH in activating cAMP activity of the I173M *MC4R* (0.05 ± 0.02µM) was significantly lower than that of the WT *MC4R* (0.46 ± 0.07µM) (Table 1). It turns out that M173 *MC4R* increased ligand-induced cAMP signaling activation.

**Table 1:** The signaling properties of WT and mutant *MC4R*s in response to ligand stimulation

Ligand		cAMP Response		MAPK/ERK Response	
		EC_50_ (µM)	Rmax(%WT)	EC_50_ (µM)	Rmax(%WT)
α-MSH	WT	0.13 ± 0.02	100.0 ± 7.1	0.98 ± 0.03	100.0 ± 3.2
	M173	0.03 ± 0.01	122.4 ± 2.1	0.26 ± 0.03	116.2 ± 2.1
	*P* value	0.009	0.0001	0.004	0.227
β-MSH	WT	0.46 ± 0.07	100.1 ± 1.2	5.61 ± 2.16	100.0 ± 5.2
	M173	0.05 ± 0.02	104.5 ± 1.0	3.59 ± 1.21	138.7 ± 2.4
	*P* value	0.0003	0.067	0.547	0.001

### The I173M mutation in *MC4R* mediates enhanced MAPK/ERK signal activation

*MC4R* is known to activate the p44/42 mitogen-activated protein kinases (MAPK), also referred to as extracellular signal-regulated kinases 1 and 2 (ERK1/2). The activation of the ERK1/2 pathway is considered a potential cellular mechanism involved in regulating *MC4R* [46–48].

To evaluate the activation of MAPK/ERK by both the WT and I173M mutant forms of *MC4R*, we employed a dual-luciferase reporter gene experiment using the ERK pathway reporter gene vector pSRE-luc. HEK293T cells were transfected with pGL4.33, pEGFP-N1, and either WT or M173 *MC4R*, followed by stimulation with six concentrations of α-MSH/β-MSH (Fig. 5a). Both receptors exhibited dose-dependent responses to varying concentrations of α-MSH/β-MSH (Fig. 5i and l). Analysis of EC_50_ values revealed that the M173 *MC4R* enhanced ligand-induced MAPK/ERK signaling activation. Specifically, the EC_50_ value of α-MSH in activating the M173 *MC4R* (0.26 ± 0.03µM) was significantly lower than that of the WT *MC4R* (0.98 ± 0.03µM) (*P* < 0.05, Fig. 5j). This indicated a fourfold increase in activation efficiency, while the Rmax value did not show significant change (Fig. 5k). There was no significant difference in EC_50_ values upon stimulation with β-MSH in terms of MAPK/ERK (Fig. 5m). However, Rmax value of the M173 *MC4R* showed a substantial increase (*P* < 0.05, Fig. 5n). Specific EC_50_ and Rmax values was shown in Table 1.

Together, these data suggest that the sheep *MC4R* I173M mutation enhances cAMP signal transduction activation, increases receptor basal activity, and promotes the activation of MAPK/ERK signaling. The I173M mutation significantly enhances the functionality of the mutated receptor, establishing it as an important functional variant in the regulation of estrus in sheep.

### The effects of transfecting WT and M173 *MC4R* genes on the proliferation, apoptosis, and steroid secretion-related gene expression in ovarian granulosa cells

**Fig. 6:**
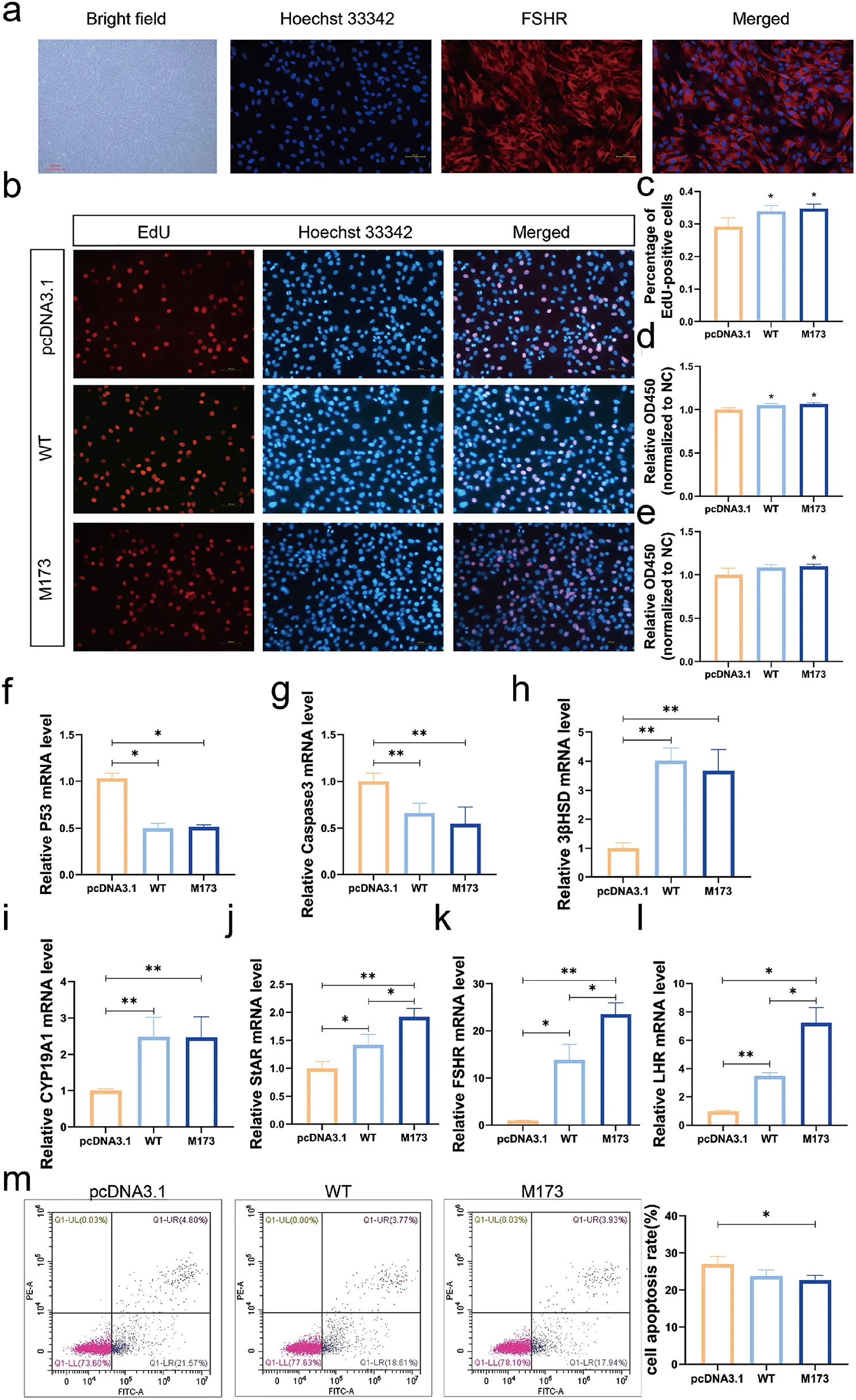
Effects of WT and I173M *MC4R* on ovarian granulosa cell function. (**a**) Immunofluorescence analysis of FSHR expression in ovarian granulosa cells. (**b**, **c**) EdU assay showing cell proliferation and quantification of EdU-positive cells in cells transfected with empty vector (pcDNA3.1), WT, or I173M MC4R. Scale bar = 100 μm. (**d**, **e**) CCK-8 assay was used to assess the viability of granulosa cells at 24 h (**d**) and 48 h (**e**) after transfection with empty vector (pcDNA3.1), WT MC4R, or M173 MC4R expression vectors. (**f**-**l**) Relative mRNA expression of *P53* (**f**), *Caspase-3* (**g**), *3βHSD* (**h**), *CYP19A1* (**i**), *StAR* (**j**), *FSHR* (**k**), and *LHR* (**l**) determined by qRT-PCR. The data were presented as the mean ± SD (*n* = 3). Statistical significance was set at *P* < 0.05 (*), *P* < 0.01 (**) or *P* < 0.001 (***). (**m**) Flow cytometry analysis of apoptotic cells

The ovarian granulosa cells exhibited characteristic polygonal/round morphology, with immunofluorescence confirming robust FSHR protein localization (Fig. 6a), validating cellular specificity and purity for functional studies. To quantify proliferative dynamics, EdU incorporation and CCK-8 assays were implemented. EdU-positive nuclei frequency revealed augmented proliferation in both WT and M173 *MC4R*-transfected cohorts, though no intergroup statistical significance was detected (Fig. 6b and c). CCK-8 kinetic profiling corroborated these trends, demonstrating sustained viability enhancement across 24–48 h post-transfection in both experimental groups (Fig. 6d and e).

Quantitative analysis of apoptotic regulators revealed coordinated downregulation of *Caspase-3* and *P53* in transfected cells relative to controls, yet no genotype-dependent differential expression was observed (Fig. 6f and g), suggesting *MC4R*-mediated antiapoptotic effects are mutation-independent. Meanwhile, steroidogenic pathway genes, 3β*HSD* (*HSD3B1*), *CYP19A1*, *StAR*, *FSHR*, and *LHR*, exhibited marked upregulation in both WT and M173 transfectants (Fig. 6h–l). Annexin V/PI double-staining followed by flow cytometry revealed a significant reduction in apoptosis in M173-transfected cells compared with controls (Fig. 6m, *P* < 0.05). Mechanistically, M173 variants potentiated transcriptional activation of *FSHR*, *LHR*, and *StAR* beyond WT levels (*P* < 0.05), implicating this missense mutation in hyperactivation of gonadotropin-responsive steroidogenic networks.

## Discussion

**Fig. 7:**
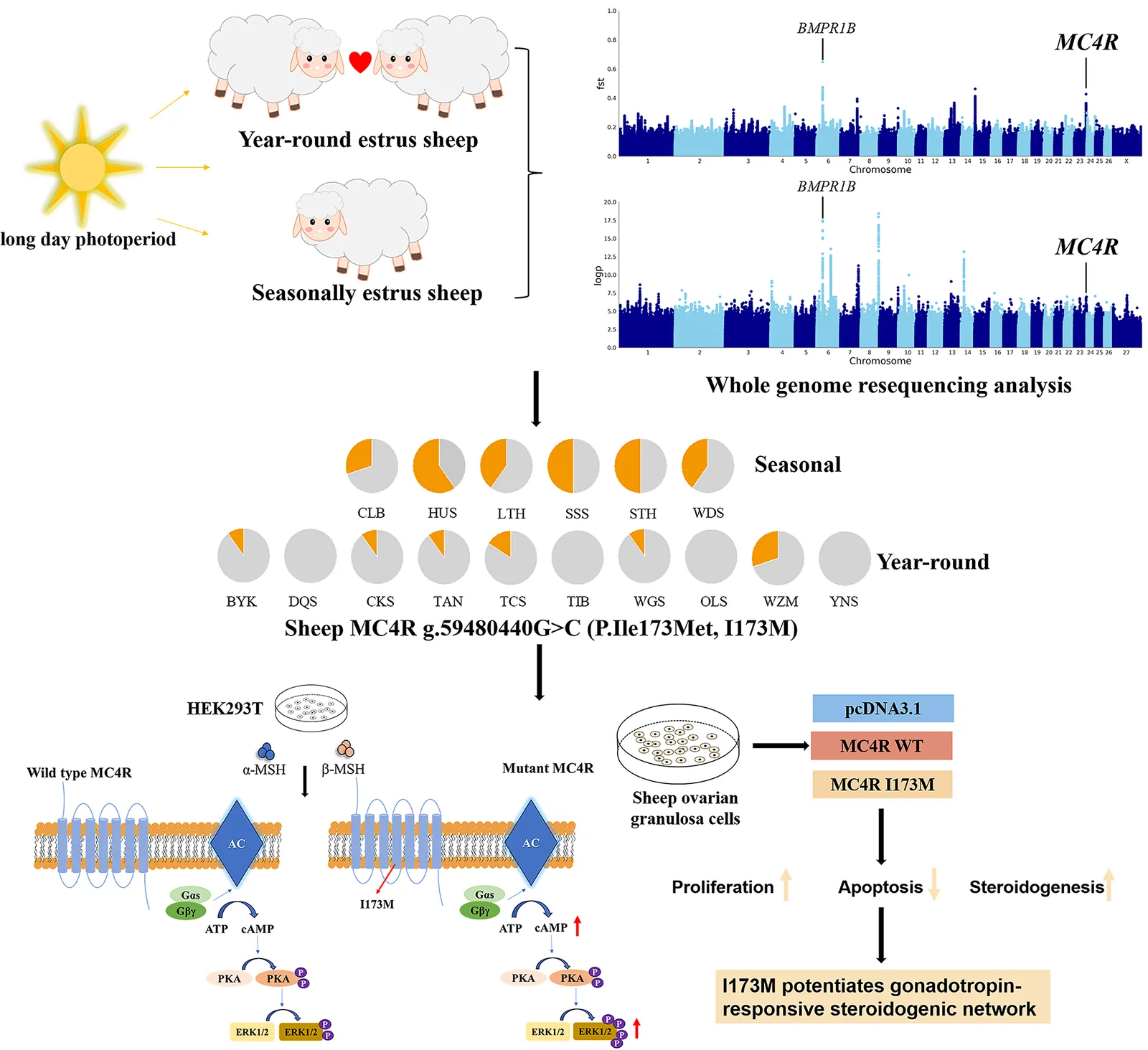
Graphical summary of the molecular mechanism underlying the gain-of-function mutation I173M in the sheep *MC4R* gene

Seasonal estrus poses a significant constraint on the reproductive capacity of sheep. Investigating the regulatory mechanisms of year-round estrus in sheep is crucial for enhancing their reproductive efficiency. Through a genomic approach, utilizing comprehensive sheep whole-genome resequencing data and estrus information, and employing methodologies like selective signal detection and association analysis, we provide strong evidence that *MC4R* is a novel candidate gene for estrus seasonality in sheep, particularly through the I173M mutation site which may potentially influence the traits associated with year-round estrus in sheep (Fig. 7).

*MC4R* is widely expressed in the brain and regulates both food intake and energy expenditure [49]. Beyond its metabolic role, *MC4R* has been shown to play a crucial role in reproductive function by regulating LH release, ovulation, and fertility [40, 50]. The C allele of rs571312 and the G allele of rs12970134 in the *MC4R* gene have been found to be linked with precocious puberty among girls suffering from obesity [51]. Previous studies have revealed disruptions in follicular dynamics and a reduction in corpus luteum count in *MC4R* knockout mice. The regularity of the estrous cycle in mice is significantly influenced by genetic factors, as evidenced by the pronounced impact of *MC4R* knockout on the rhythmicity of the mouse estrous cycle [52, 53]. Similarly, in rats, *MC4R* has been shown to impact the secretion of luteinizing hormone (LH), potentially influencing the estrous cycle [40]. Quantitative analysis of *MC4R* expression in the hypothalamus of female rats at different estrous stages indicates elevated expression during the pre-estrus phase, followed by downregulation during estrus, post-estrus, and diestrus [54]. LH secretion is stimulated by α-MSH in male humans [55], whereas in rats, the administration of *MC4R* antagonists reduces the magnitude of the LH surge [56]. Reinsertion of Mc4r in Kiss1 neurons of MC4R null mice restores estrous cyclicity and LH pulsatility, highlighting the critical role of MC4R in Kiss1-mediated neuroendocrine regulation [53]. Moreover, *MC4R* signaling stimulates GnRH expression in the hypothalamus, thereby modulating the synthesis of FSH and LH in the pituitary [57]. These results suggest that the upregulation of *MC4R* expression may be associated with the pre-ovulatory surge in GnRH and LH, further substantiating the involvement of *MC4R* in the regulation of the animal’s estrous cycle. In summary, these findings suggest that *MC4R* is involved in the regulation of reproduction in mammals through neuroendocrine control. Since reproductive processes are highly energy-dependent, particularly in seasonal breeders such as sheep, disruption of MC4R signaling may impair the energy–reproduction axis. Although studies in sheep have so far linked *MC4R* mainly to growth traits, meat quality, and feed intake [58–60], the co-localization of MC4R and kisspeptin in the arcuate nucleus (ARC) [61], together with the known involvement of the KISS1–GPR54/TSHR–DIO2/DIO3 pathway in year-round estrus [3], suggests that MC4R may also play a role in promoting non-seasonal estrus in sheep.

Previous studies have shown that *MC4R* expressed in HEK293T cells can be stimulated to activate transcription by melanocortin analogues at different concentrations, allowing exploration of its regulation of downstream signaling pathways [62, 63]. Given the high degree of conservation between sheep MC4R and human MC4R protein, we conducted further research on the regulatory effects of sheep *MC4R* gene I173M mutation in HEK293T cells. Our analysis shows that the variant does not affect total protein expression and receptor internalization. Transcriptome sequencing results revealed that the biological processes of signaling receptor ligand activity and receptor activator activity were enriched, and cAMP signaling pathway and MAPK signaling pathway were significantly enriched after transfected with M173 *MC4R* plasmid. In consistency with these results, our study identified *MC4R* gene I173M mutation as a functionally acquired mutation, enhancing the basal activity of the mutated receptor and upregulating the cAMP and MAPK/ERK signaling pathways. The cAMP signaling regulates follicle growth, meiosis, and ovulation [64]. Additionally, reproduction-related pathways, such as GnRH signaling pathway and ovarian steroidogenesis pathways, were significantly enriched, suggesting an influence on the hypothalamic-pituitary-ovarian axis and influencing hormone secretion in animals. Furthermore, previous research has suggested that obesity can affect various aspects of female reproductive capability, including oocyte growth and development, ovulation, endometrial growth, embryo development, and embryo implantation, ultimately influencing estrus [65–67]. Previous studies have shown that the *MC4R* gene I173M mutation can significantly increase gene transcriptional activity [68]. The I173M mutation has been associated with the adult height of Hu sheep and has effects on gene transcriptional activity, with the homozygous wild type linked to higher body weight [68]. Functional gain-of-function mutations in *MC4R* can prevent obesity and exhibit favorable metabolic characteristics, potentially serving as crucial factors influencing sheep estrus. Therefore, the I173M mutation may impact hormone secretion and energy metabolism, affecting sheep estrus.

This study leverages sequencing data from both year-round and seasonal estrus sheep, establishing a significant association between the novel candidate gene *MC4R* and its I173M mutation with estrus traits in sheep. Significantly, it marks the first exploration of the cellular mechanisms underlying the I173M mutation in the sheep *MC4R* gene. Mechanistically, this mutation induced an augment basal activity of the *MC4R* receptor, enhancing the efficiency of binding between the mutated receptor and agonists (α-MSH, β-MSH) is enhanced. This indicates a functionally acquired mutation that may strengthen the regulatory role of the *MC4R* receptor in estrus. While recognizing the pivotal role of the *MC4R* gene in year-round estrus in sheep, it is crucial to acknowledge that it is not the sole determination of this reproductive trait. Other contributing elements such as other genes [17], hormone levels [69], and nutritional status [70] collectively impact estrus traits in sheep. Given the complexity of estrus traits, our current study lacks direct evidence of *MC4R* regulating sheep estrus. Further research and functional validation of *MC4R* are essential to gain additional insights. Potential ways could include experiments to detect hormone levels after activating *MC4R* receptors in vivo, overexpressing the *MC4R* gene in sheep ovarian granulosa cell lines to explore its function, and conducting transcriptomic and metabolomic sequencing analyses on tissues from sheep with different genotypes of the I173M mutation. After supplementing these experiments, the regulatory role of the *MC4R* gene and its I173M mutation in the year-round estrus of sheep will become more concrete.

To better understand the evolutionary origin of the I173M variant, we investigated its distribution across wild sheep (Mouflon) and domestic sheep populations. The I173M variant was absent in all 33 Mouflon samples analyzed in this study, suggesting that it most likely arose after sheep domestication rather than being inherited from wild ancestors. Although the variant was identified in multiple geographically distinct domestic sheep breeds, a shared ancestral origin may be more plausible than multiple independent mutational events, considering the relatively low likelihood of identical missense mutations arising independently at the same genomic position. This interpretation is further supported by the observed distribution pattern of the variant, which was detected in several domestic sheep breeds, with relatively higher allele frequencies in year-round estrous breeds and lower frequencies in a few foreign breeds (Additional file 10, Fig S3). Nevertheless, the current evidence is insufficient to definitively distinguish between these two evolutionary scenarios. Future analyses incorporating haplotype structure and linkage disequilibrium will be required to clarify the evolutionary history of this variant.

Our investigation systematically delineated the functional divergence between WT *MC4R* and its I173M variant in modulating ovarian granulosa cell dynamics. Quantitative assessments via EdU and CCK-8 assays demonstrated that transfection with either WT or mutant *MC4R* constructs significantly enhanced proliferative indices and cellular viability compared to controls. Our RT-qPCR results revealed coordinated downregulation of apoptotic mediators *Caspase-3* and *P53*, mechanistically linking *MC4R* activation to prosurvival signaling pathways critical for follicular maintenance. Notably, *MC4R* overexpression induced pronounced upregulation of steroidogenic effectors (*3βHSD*, *CYP19A1*, *StAR*) and gonadotropin receptors (*FSHR*, *LHR*), with the I173M variant exhibiting gain-of-function properties: *FSHR*, *LHR*, and StAR mRNA levels in mutant transfectants surpassed WT counterparts. Given *StAR’s* gatekeeping role in cholesterol transport [71] and *CYP19A1’s* enzymatic dominance in estrogen biosynthesis, these data implicate *MC4R* as a transcriptional rheostat coordinating ovarian steroidogenesis. Furthermore, elevated *FSHR*/*LHR* expression suggests *MC4R* potentiates gonadotropin signaling sensitivity, a prerequisite for follicular maturation and ovulation [72]. While *MC4R* is canonically characterized as a hypothalamic energy homeostasis regulator [73, 74], our findings establish its *de novo* role in peripheral reproductive circuits. Specifically, *MC4R* is suggested to modulate ovine estrous cyclicity through dual mechanisms: (1) amplifying steroidogenic output via *StAR*/*CYP19A1* induction, and (2) sensitizing follicular responses to gonadotropins through *FSHR*/*LHR* upregulation. This functional pleiotropy positions *MC4R* as a critical node bridging metabolic signaling and reproductive competence, with the hyperactive I173M variant likely conferring mutation-specific hyperactivity in steroidogenic pathways, an evolutionary adaptation underlying constitutive estrous activity in prolific sheep breeds.

This study provides the first functional evidence of the *MC4R* I173M mutation in sheep, but several limitations should be acknowledged. Due to the considerable challenges of conducting in vivo functional studies in sheep, our functional validation was primarily based on in vitro experiments and lacks in vivo confirmation. Moreover, the breeds analyzed may not fully represent population diversity, and the unbalanced sample sizes across groups may introduce potential population structure effects on GWAS and FST results. And estrous regulation involves multiple genes and environmental factors beyond *MC4R*. Nevertheless, despite these limitations, these findings not only deepen the understanding of the genetic mechanisms underlying seasonal and year-round estrus but also suggest that *MC4R* I173M serves as a promising candidate marker for marker-assisted breeding.

## Conclusions

In summary, our results provide new evidence supporting a previously unrecognized role of the *MC4R* gene in regulating estrus seasonality in sheep. The I173M mutation is strongly associated with year-round estrus traits, offering novel insights into *MC4R* function and identifying a potential target for enhancing reproductive performance in seasonally estrous breeds. These findings advance our understanding of the genetic regulation of estrus and suggest that the *MC4R* I173M variant could be leveraged in future breeding programs to improve reproductive efficiency and facilitate molecular breeding strategies. 

## Supplementary Information

Below is the link to the electronic supplementary material.

Supplementary Material 1 Additional file 1, Table S1 Format: word Title: Table S1. Sample information Description: Sample information.

Supplementary Material 2 Additional file 2, Table 2 Format: Excel (.xlsx) Title: Table S2. Metadata for whole-genome sequencing samples. Description: Information on the whole-genome sequencing samples used in this study, including sample ID, run accession, breed, origin region, origin, average sequencing depth, and data source.

Supplementary Material 3 Additional file 3, Table S3 Format: word Title: Table S3. qPCR primer information table Description: qPCR primer information table.

Supplementary Material 4 Additional file 4, Fig S1 Format: word Title: Fig S1. Genetic variation analysis of MC4R among sheep samples. (**a**) PCA analysis among samples in this experiment. (**b**) Heatmap showing SNP frequency in theMC4R gene across 16 sheep breeds. Description: Genetic variation analysis of MC4R among sheep samples.

Supplementary Material 5 Additional file 5, Fig S2 Format: word Title: Fig S2. Model-based clustering of sheep breeds using ADMIXTURE with K = 5 (CV =0.302). K = 5 is the lowest value of CV error. Description: Model-based clustering of sheep breeds.

Supplementary Material 6 Additional file 6, Table S4 Format: Excel (.xlsx) Title: Table S4. A summary of genes from FST (top1%). Description: A summary of genes from FST (top1%).

Supplementary Material 7 Additional file 7, Table S5 Format: Excel (.xlsx) Title: Table S5. A summary of genes from GWAS (top1%). Description: A summary of genes from GWAS (top1%).

Supplementary Material 8 Additional file 8, Table S6 Format: word Title: Table S6. SNP information within MC4R gene in 16 breeds Description: SNP information within MC4R gene in 16 breeds.

Supplementary Material 9 Additional file 9, Table S7 Format: Excel (.xlsx) Title: Table S7. SNP frequency in the MC4R gene across 16 sheep breeds Description: SNP frequency in the MC4R gene across 16 sheep breeds.

Supplementary Material 10 Additional file 10, Fig S3 Format: word Title: Fig S3. Allele frequencies of the MC4R I173M (g.59480440G>C, p.Ile173Met) variant in Mouflon and different domestic sheep breeds. Description: Allele frequencies of the MC4R I173M (g.59480440G>C, p.Ile173Met) variant in Mouflon and different domestic sheep breeds.

## Data Availability

The original contributions presented in the study are included in the article/Supplementary Material. Further inquiries can be directed to the corresponding authors upon reasonable request.
