# Supplementary material for: Cellular mechanism of gain-of-function mutation I173M in sheep MC4R gene identified in year-round and seasonal estrus breeds through whole-genome resequencing
Source: Genet Sel Evol. 2026 Oct 12;58:72. doi: 10.1186/s12711-026-01091-4 (PMC13656466; doi:10.1186/s12711-026-01091-4)
Supplement: Supplementary file 1 [file 12711_2026_1091_MOESM1_ESM.docx]

**Table S1. Sample information**

| **Breeds** | **Abbreviations** | **Estrus type** | **Number** |
| --- | --- | --- | --- |
| Cele black sheep | CLB | year-round estrus | 40 |
| Hu sheep | HUS | year-round estrus | 50 |
| Large Tail Han sheep | LTH | year-round estrus | 12 |
| Sishui fur sheep | SSS | year-round estrus | 11 |
| Small Tail Han sheep | STH | year-round estrus | 29 |
| Wadi sheep | WDS | year-round estrus | 18 |
| Bayinbuluke sheep | BYK | seasonal estrus | 31 |
| Diqing sheep | DQS | seasonal estrus | 13 |
| Chaka sheep | CKS | seasonal estrus | 10 |
| Tan sheep | TAN | seasonal estrus | 38 |
| Tengchong sheep | TCS | seasonal estrus | 9 |
| Tibetan sheep | TIB | seasonal estrus | 50 |
| Waggir Sheep | WGS | seasonal estrus | 10 |
| Oula sheep | OLS | seasonal estrus | 16 |
| Wuzhumuqin sheep | WZM | seasonal estrus | 10 |
| Yunnan sheep | YNS | seasonal estrus | 45 |
