# Supplementary material for: Cellular mechanism of gain-of-function mutation I173M in sheep MC4R gene identified in year-round and seasonal estrus breeds through whole-genome resequencing
Source: Genet Sel Evol. 2026 Oct 12;58:72. doi: 10.1186/s12711-026-01091-4 (PMC13656466; doi:10.1186/s12711-026-01091-4)
Supplement: Supplementary file 2 [file 12711_2026_1091_MOESM3_ESM.docx]

| **Gene** | **Forward primer** | **Reverse primer** |
| --- | --- | --- |
| *LHR* | GCTGAGGGCTACCAACAAAG | GCAGCTGAGATGGCAAAGAA |
| *FSHR* | CTCAGTGTGGAGCCTCTGAA | GCATCTCTGTCACCTTGCTG |
| *3βHSD* | CAGTCTATGTTGGCAATGTGGC | CGGTTGAAGCAGGGGTGGTAT |
| *STAR* | ATTCAGGAGGCAAAGAGCAGC | TCGGGTAAGGAAAATGGGTCA |
| *CYP19A1* | TCGTCCTGGTCACCCTTCTG | CGGTCTCTGGTCTCGTCTGG |
| *Caspase3* | TTTCAGAGGGGACTGTTGCAG | CAGTCCAGTTCTGTGCCTCG |
| *P53* | TTCCCCTTCCCTCAACAAGC | CCTCACAACCTCCGTCATGT |
| *GAPDH* | TGACCTGCCGCCTGGAGAAA | GTAGAAGAGTGAGTGTCGCTGTT |

**Table S3. PCR primer information table**
