# Supplementary material for: Cellular mechanism of gain-of-function mutation I173M in sheep MC4R gene identified in year-round and seasonal estrus breeds through whole-genome resequencing
Source: Genet Sel Evol. 2026 Oct 12;58:72. doi: 10.1186/s12711-026-01091-4 (PMC13656466; doi:10.1186/s12711-026-01091-4)
Supplement: Supplementary file 3 [file 12711_2026_1091_MOESM4_ESM.docx]

Fig S1. Genetic variation analysis of *MC4R* among sheep samples.

(a) PCA analysis among samples in this experiment. (b) Heatmap showing SNP frequency in the *MC4R* gene across 16 sheep breeds.
