# Supplementary material for: Cellular mechanism of gain-of-function mutation I173M in sheep MC4R gene identified in year-round and seasonal estrus breeds through whole-genome resequencing
Source: Genet Sel Evol. 2026 Oct 12;58:72. doi: 10.1186/s12711-026-01091-4 (PMC13656466; doi:10.1186/s12711-026-01091-4)
Supplement: Supplementary file 4 [file 12711_2026_1091_MOESM5_ESM.docx]

Fig S2. Model-based clustering of sheep breeds using ADMIXTURE with K = 5 (CV = 0.302). K = 5 is the lowest value of CV error.
