# Supplementary material for: Cellular mechanism of gain-of-function mutation I173M in sheep MC4R gene identified in year-round and seasonal estrus breeds through whole-genome resequencing
Source: Genet Sel Evol. 2026 Oct 12;58:72. doi: 10.1186/s12711-026-01091-4 (PMC13656466; doi:10.1186/s12711-026-01091-4)
Supplement: Supplementary file 5 [file 12711_2026_1091_MOESM8_ESM.docx]

**Table S6. SNP information within MC4R gene in 16 breeds**

| Chromosome | Positions | Regions | Mutation types | Ref | Alt |
| --- | --- | --- | --- | --- | --- |
| 23 | 59477500 | 3′ UTR | - | G | A |
| 23 | 59477576 | 3′ UTR | - | G | A |
| 23 | 59477599 | 3′ UTR | - | A | G |
| 23 | 59477643 | 3′ UTR | - | A | G |
| 23 | 59477651 | 3′ UTR | - | C | A |
| 23 | 59477711 | 3′ UTR | - | G | A |
| 23 | 59477718 | 3′ UTR | - | C | T |
| 23 | 59477758 | 3′ UTR | - | A | G |
| 23 | 59477796 | 3′ UTR | - | A | G |
| 23 | 59477848 | 3′ UTR | - | A | G |
| 23 | 59477852 | 3′ UTR | - | A | G |
| 23 | 59477889 | 3′ UTR | - | A | G |
| 23 | 59477958 | 3′ UTR | - | C | T |
| 23 | 59477959 | 3′ UTR | - | G | A |
| 23 | 59477991 | 3′ UTR | - | A | G |
| 23 | 59478305 | 3′ UTR | - | G | C |
| 23 | 59478417 | 3′ UTR | - | C | T |
| 23 | 59478659 | 3′ UTR | - | A | G |
| 23 | 59478669 | 3′ UTR | - | A | G |
| 23 | 59478815 | 3′ UTR | - | T | C |
| 23 | 59479003 | 3′ UTR | - | A | G |
| 23 | 59479018 | 3′ UTR | - | A | C |
| 23 | 59479076 | 3′ UTR | - | A | G |
| 23 | 59479150 | 3′ UTR | - | G | C |
| 23 | 59479421 | 3′ UTR | - | G | A |
| 23 | 59479640 | 3′ UTR | - | G | A |
| 23 | 59479719 | 3′ UTR | - | A | G |
| 23 | 59479943 | 3′ UTR | - | C | T |
| 23 | 59480440 | exon | nonsynonymous | G | C |
| 23 | 59480866 | exon | synonymous | C | T |
| 23 | 59480947 | exon | synonymous | G | C |
| 23 | 59480950 | exon | synonymous | G | A |
| 23 | 59481164 | 5′ UTR | - | C | T |
| 23 | 59481245 | 5′ UTR | - | C | T |
