# Supplementary material for: Cellular mechanism of gain-of-function mutation I173M in sheep MC4R gene identified in year-round and seasonal estrus breeds through whole-genome resequencing
Source: Genet Sel Evol. 2026 Oct 12;58:72. doi: 10.1186/s12711-026-01091-4 (PMC13656466; doi:10.1186/s12711-026-01091-4)
Supplement: Supplementary file 6 [file 12711_2026_1091_MOESM10_ESM.docx]

Fig S3. Allele frequencies of the MC4R I173M (g.59480440G>C, p.Ile173Met) variant in Mouflon and different domestic sheep breeds
